# Towards low energy greywater treatment of surfactants and pathogens

**DOI:** 10.1007/s43832-025-00295-x

**Published:** 2025-10-28

**Authors:** Zachary Bogart, Aksana Atrashkevich, Jirapat Ananpattarachai, Shahnawaz Sinha, Sergi Garcia-Segura, Paul Westerhoff

**Affiliations:** School of Sustainable Engineering and the Built Environment, Arizona State University, Tempe, AZ 85287-3005 USA

**Keywords:** Foam fractionation, Greywater, Water reuse, Water, Water treatment

## Abstract

Greywater reuse in households and off-grid applications is growing in response to water scarcity. Effective treatment technologies must balance water quality, efficiency, and low energy or chemical inputs. This study presents a low-energy, non-biological greywater treatment system using foam fractionation and in situ electrochlorination, leveraging ambient chloride in greywater. Designed for handwashing stations and household reuse, the system treats up to 450 L day^− 1^ using < 1 kWh day^− 1^ of energy with no chemical dosing. Among six screened physical-chemical processes, foam fractionation was the only method achieving > 80% COD removal with high water recovery. Smaller diffuser pore sizes and lower air flowrates improved COD removal by increasing bubble surface area, though at the cost of water recovery. Pulsed aeration improved water recovery by ~ 20% with minimal COD performance loss. Surfactant behavior, correlated with the critical micelle concentration (CMC), strongly influenced foam stability and COD removal. Both free and combined chlorine residuals were successfully generated via in situ electrochlorination, using only the chloride ions naturally present in greywater. In situ electrochlorination following foam treatment generated up to 0.53 mg L^− 1^ of residual chlorine. Foam fractionation with small-pore diffusers removed surfactants and other compounds that inhibit chlorine generation, such as CAPB, SDS, and anions, improving disinfection outcomes. The system achieved > 70% COD removal and > 80% water recovery. This integrated, chemical-free system provides a promising solution for decentralized greywater reuse. With further optimization, it may consistently achieve < 50 mg L^− 1^ COD and adequate disinfection, supporting sustainable hygiene in low-resource settings.

**Supplementary Information:**

The online version contains supplementary material available at 10.1007/s43832-025-00295-x.

## Introduction

Population growth and climate change are intensifying global water scarcity [1]. In households, only about 1% of domestic water is used for cooking or drinking, while 50–70% goes toward showers, baths, washing machines, and bathroom sinks—all sources of greywater [2]. The remaining 30–50%, primarily from toilets and kitchens, is classified as blackwater due to its higher organic and microbial content [3]. To ease pressure on potable water supplies, both developed and developing countries are increasingly reusing greywater. The required treatment level depends on the reuse application, with toilet flushing—due to its high demand—and outdoor irrigation being the most common [4]. There is a growing need for affordable, low-maintenance, and easily deployable greywater treatment technologies [5].

Greywater reuse is occurring globally [6, 7]. In Australia, greywater is reused for most household activities where non-potable water is suitable [8, 9]. In contrast, Germany more commonly relies on rainwater harvesting for toilet flushing than on domestic-scale greywater reuse. In the United States, greywater reuse is being integrated into State-level regulations, but accounts for a small share of total domestic water use. In many developing regions, inadequate sanitation infrastructure remains a major barrier. Greywater reuse offers a potential solution: for instance, a recent study in Ghana found that 18% of households regularly reuse laundry greywater [10]. Public acceptance of non-potable greywater reuse appears relatively high. However, broader adoption is often constrained by concerns about public health, cost, reliability, and maintenance—especially when municipal tap water remains more convenient [2, 6, 11].

There are a broad range of natural, biological, and physicochemical treatment processes capable of producing greywater suitable for toilet flushing, handwashing, and outdoor irrigation [3, 5, 12–15]. These technologies are typically evaluated at the household or apartment-building scale, but even smaller-scale applications are possible [16]. Handwashing stations in transit hubs, schools, sports venues, and clinics also generate greywater, and in-situ treatment at these sites could enable reuse while reducing water waste [15, 17]. Recent studies have explored handwashing water recycling systems using bioreactors and biochar [15, 18]. However, relatively few portable greywater systems are tailored specifically for handwashing stations [15]. Reusing water in these settings—especially in high-traffic or resource-limited areas—offers multiple benefits: conserving water, supporting off-grid clinics and emergency deployments, maintaining station functionality in water-stressed regions, encouraging frequent handwashing, minimizing stagnant wastewater that can attract disease vectors, and raising public awareness of sustainable water use. This is particularly valuable as a low-cost educational tool in schools and communities. Accordingly, this work seeks to identify which residential greywater treatment technologies can be adapted for small-scale, low-energy handwashing applications.

Greywater contains surfactant soaps, salts, pathogens, oils, skin cells, hair, and inorganic colloids [19–21]. While particulate matter affects both pathogen load and aesthetic quality, the removal of chemical oxygen demand (COD) and associated organics remains a key challenge for greywater treatment. Surfactants are major contributors to COD and significantly influence treatability. There are four main types: anionic, cationic, zwitterionic, and nonionic. All possess both hydrophobic and hydrophilic ends, enabling them to interact with water and oils. Anionic surfactants—the most abundant—are the primary cleaning agents in household soaps and effectively remove a wide range of particles [22]. Cationic surfactants, commonly found in conditioners, reduce foaming and neutralize hair charge. Zwitterionic surfactants, often used in body washes, are gentler on the skin and boost foaming when paired with anionic types. Nonionic surfactants, typically used in laundry detergents, are especially effective at dissolving oils. Table 1 summarizes relevant reuse standards for sinks and toilets [23].

**Table 1:** Representative Greywater composition, treatment goals to Meet reuse standards for toilet Flushing and/or handwashing [23], and model Greywater ingredients used in experiments

Greywater Parameters of Interest	Treatment Goal Category A - Unrestricted urban uses	Treatment Goal Category B - Restricted urban uses	Spiked pollutant concentration for model greywater used in experiments
Chemical Oxygen Demand (COD) Biochemical oxygen demand (BOD)	≤ 50 mg L^− 1^ ≤ 10 mg L^− 1^	≤ 150 mg L^− 1^ ≤ 10 mg L^− 1^	Surfactants: 0.22 mM cocamidopropyl betaine (CAPB); 1.0 mM sodium dodecyl sulfate (SDS); 0.23 mM hydroxyethylcellulose ethoxylate (HECE) 100 mg L^− 1^ Cellulose 100 mg L^− 1^ Yeast extract
Total suspended solids (TSS)	≤ 10 mg L^− 1^	≤ 30 mg L^− 1^	100 mg L^− 1^ kaolin
Total nitrogen	≥ 70% reduction or ≤ 15 mg L^− 1^		0.74 mM Ammonium bicarbonate 0.32 mM Sodium nitrate
Total phosphorus	≥ 80% reduction or ≤ 2 mg L^− 1^		0.31 mM Sodium phosphate
pH	6 to 9		8 (controlled only by salts and surfactants added)
Colour	≤ 30 pcu		--
Conductivity or Total Dissolved Solids (TDS)	No limit		Other salts & hardness ions (3.1 mM sodium chloride; 0.5 mM calcium chloride; 0.4 mM magnesium sulfate)
Indicator microorganisms in effluent	Bacteria: *E. coli* ≥ 6 LRV Viruses: *MS2 Coliphage* ≥ 7 LRV Helminths: *Ascaris suum viable ova* ≥ 4 LRV Protozoa: *viable Clostridium perf. spores* ≥ 6 LRV		We only considered disinfection of *E. coli* and *MS2* using electrochlorination, based on the previous work that employed “Micro Flow Cell” setup from ElectroCell (Sweden), which is the same setup used in the current study [24].

This study aimed to develop a low-energy and low-chemical demanding non-biological greywater treatment system capable of meeting water quality standards with high water recovery. Designed to treat 450 L day^− 1^ of household greywater using under 1 kWh day^− 1^ of electricity and minimal chemicals, the system followed a Bill and Melinda Gates Foundation request discouraging biological processes due to past reliability and maintenance issues. We considered and screened various adsorption, precipitation, and oxidation methods, but only foam fractionation met the treatment goals. Therefore, subsequent research investigated key operational parameters of foam-based treatment—such as diffuser type, gas flow rate (affecting bubble size), and foam management strategies. We also assessed the foaming behavior of individual and mixed surfactants, both in terms of removal and dependence of bubble formation on critical micelle concentration (CMC). As the project evolved, the Foundations focus shifted to smaller-scale applications (e.g., handwashing stations < 50 L day^− 1^). To ensure safe reuse, we integrated in-situ electrochlorination and examined how foam-fractionated greywater chemistry influenced chlorine speciation and dose efficacy.

## Methodology

### Greywater composition

Although greywater composition varies widely, we used a formulation in our experiments that reflects concentrations and constituents commonly reported in the literature [21]. The resulting initial COD was ~ 1000 mg L^− 1^. To simulate common surfactants in greywater, SDS, CAPB, and HECE were used to represent anionic, zwitterionic, and nonionic surfactants, respectively. Cellulose, kaolin, and yeast extract were added to mimic dead skin, dirt, and other organic particulates. Salts were included to replicate typical tap water conditions. The treatment goal was initially to achieve target COD and turbidity levels (< 50 mg L^− 1^ and < 2 NTU, respectively), while nutrient removal was a lower priority during initial technology screening. Because we recently published detailed studies on electrochlorination using ambient chloride concentrations and ability to disinfect *E. coli* and MS2 bacteriophage [24–26], herein, we focus on the influence of greywater components on the electrochlorination process and production of free and combined chlorine residuals.

The following chemicals were used for the synthetic greywater: Sigma-Aldrich kaolin 795,453, Sigma-Aldrich cellulose 435,236, Sigma-Aldrich sodium dodecyl sulfate L3771, Sigma-Aldrich hydroxyethylcellulose ethoxylate, quaternized 525,944, Sigma-Aldrich sodium nitrate 221,341, Sigma-Aldrich ammonium bicarbonate A6141, Sigma-Aldrich magnesium sulfate hepta-hydrate 230,391, Sigma-Aldrich sodium chloride 59,625, Millipore yeast extract granulated 1.03753, VWR calcium chloride dihydrate 0556, VWR sodium phosphate BDH9296, and Combi-Blocks cocamidopropyl betaine NA-0048. For commercial hand soap, LACURA^®^ clear hand soap, which utilizes sodium laureth sulfate and cocamidopropyl betaine as surfactants, was chosen because of the similar surfactants to the synthetic greywater and low cost.

### Screening experiments using multiple physical-chemical processes

Six nonbiological technology approaches were screened in batch experiments to assess their ability to meet water quality goals (COD and turbidity), while minimizing energy and chemical inputs and maximizing water recovery. Screening was conducted without pH adjustment using a simplified greywater formula containing 300 mg L^− 1^ SDS and 58.5 mg L^− 1^ ammonium bicarbonate. Table 2 identifies the six treatment processes and key operational parameters, with additional details provided in Supporting Information (**Text S1**). Coagulation, flocculation and sedimentation was simulated using a jar test apparatus (Phipps & Bird PB-900 Series). Adsorption used powdered activated carbon (PAC) in the jar test apparatus with 24 h contact time. Photocatalysis used P25 TiO_2_ at 1 and 5 g L^− 1^ under UV-C irradiation (Viqua S330RL UV Lamp). Electrocatalysis used Boron-doped diamond (BDD) electrodes at 21 V and 3.5 A (33–35 mA cm^− 2^). Ultrafiltration used Pall Membralox ceramic membranes with pore sizes of 5 μm, 0.1 μm, and 20 nm under variable pressures (1.2–4.1 bar). Foam fractionation was conducted in a 5 L graduated cylinder with 500 mL of synthetic greywater, a 60 μm pore diffuser, and an air flow rate of 0.1 L min^− 1^ for up to 2 h.

### Evaluation of foam fractionation reactors and operations

**Fig. 1:**
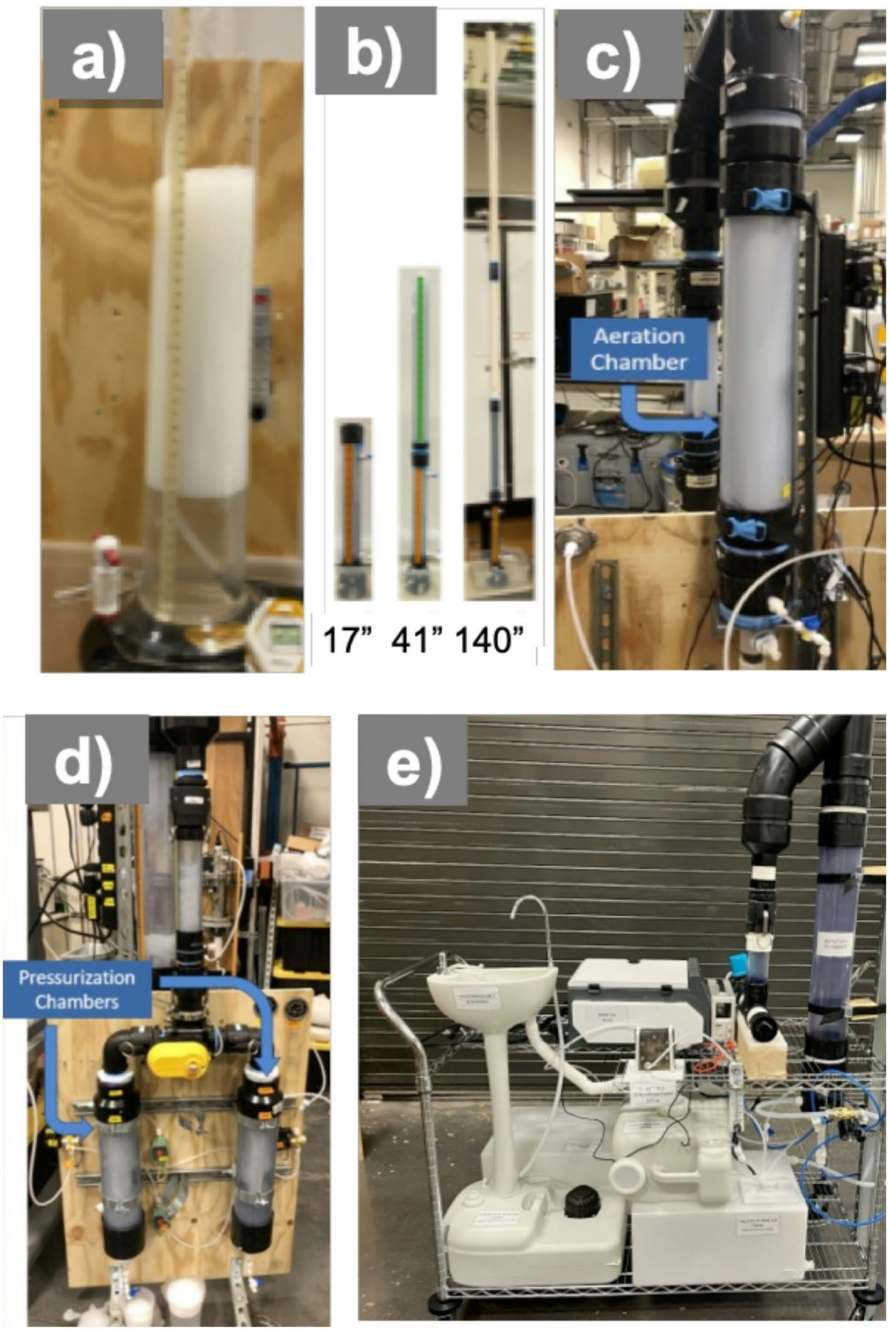
Photographs and schematics of 4 small-scale batch foam fractionation systems used for **a** screening experiments, **b** preliminary design experiments with foam rise potential of 40 to 350 cm, **c** experiments for generation 1 design with **d** integrated foam processing, and **e** experiments for generation 2 design with real greywater and integrated handwashing station and electrochlorination

**Fig. 2:**
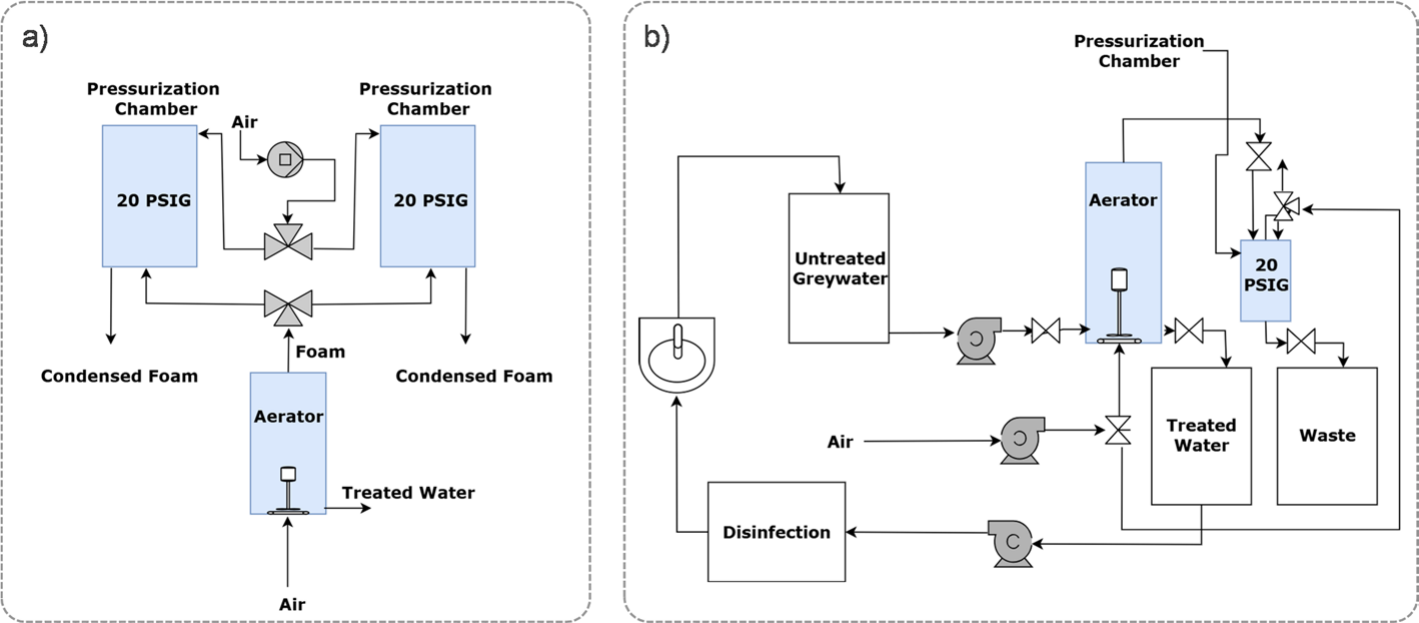
Schematics of foam fractionation system for **a** continuous flow with dual-foam collection tubes designed for up to 450 L day^− 1^ treatment capacity, and **b** semi-batch operation with single foam collection tube designed for a hand-wash station application

Screening experiments (Table 2) identified foam fractionation as the most promising method to meet performance and input targets (discussed in Results). Consequently, we designed and tested additional reactor configurations. Figure 1 presents photos and schematics of foam fractionation systems. Initial batch experiments (Fig. 1a) were conducted in a 2 L glass graduated cylinder (5 cm diameter × 38 cm height) with a diffuser stone at the base. Pressurized air (7.9 bar) was introduced to generate foam, which overflowed the top during operation. Treated water was collected from a spigot at the bottom and analyzed for COD and turbidity under varying water quality and operational conditions. To assess performance at longer foam residence times, a second system (Fig. 1b) used 5 cm diameter PVC columns up to 365 cm tall, also fitted with base diffusers (Fig. 2).

Figures 1c–e show two integrated systems with foam generation, separation, and collapse functionality. These used vertical foam columns (< 1 m tall) within one- or two-sided 10.16 cm diameter PVC tubes. Foam was collected, then sealed and pressurized (1.4 bar) to collapse it, concentrating COD and particulates into a smaller waste volume. Treated water volume (*V*_*eff*_) and collapsed waste volume (*V*_*foam*_) were used to calculate water recovery efficiency (WRF) as:

1 $$\:\text{W}\text{R}\text{F}\:=\:\frac{{V}_{eff}}{{V}_{foam}}\times100\%$$

Samples were analyzed for COD, turbidity, conductivity, pH, and other parameters.

### In-line electrochlorination generation and disinfection system for Greywater

The influence of initial and foam-treated greywater chemistry on in situ electrochlorination and disinfection potential was investigated. The method shares the same benefits as chemical chlorination, such as providing chlorine residuals that enable primary and secondary water disinfection [27]. The major advantage of electrochemical disinfection is its easy operation in decentralized settings without the need to handle hazardous chemicals. A recent study by Atrashkevich et al. has shown that in situ electrochlorination is more effective at inactivating *E. coli* and MS2 compared to chemical chlorination [24, 25]. The study used a commercially available “Micro Flow Cell” from ElectroCell, which uses platinum coated titanium plate for the cathode and dimensional stable anode (DSA^®^) Ti/RuO_2_ for the anode. The experimental operating conditions used the galvanostatic operating mode with a current of 0.2 A and a flowrate of 0.3 L min^− 1^. This higher oxidation activity is attributed to the high chlorine content near the anode surface under localized acidic pH and the potential synergies of chlorine and radical species [24–26].

### Additional analytical methods

A Hanna Instruments HI991301 Portable Multiparameter Meter was used to measure pH, electroconductivity, total dissolved solids (TDS), and temperature of the samples by setting the probe in the samples. A Milwaukee MI415 Portable Turbidity Meter was used to measure the turbidity using 10 mL of sample solution in the cuvette chamber. Contact angle was measured using a Biolin Scientific Attension Theta tensiometer and a surface material of polystyrene. COD was measured using Hach Method 8000.

## Results and discussion

### Screening of physical-chemical processes for potential to Meet treatment goals

Six non-biological treatment methods were evaluated for COD removal and other treatment criteria (Table 2). Coagulation with 500 mg L^− 1^ FeCl_3_ achieved 77% COD removal but was rated low overall due to the high coagulant dose and sludge generation. Adsorption using powdered activated carbon (PAC) required a high dose (5.0 g L^− 1^) to reach > 90% COD removal. Although it met performance targets, the low adsorption capacity and associated sludge production led to a medium rating. Photocatalysis with TiO_2_ achieved only 9% COD removal and would require ~ 552 kWh to treat 450 L day^− 1^—rendering it unfeasible due to high energy demand. Electrocatalysis achieved 65% COD removal but would require ~ 57 kWh for 450 L, indicating low potential due to slow kinetics and high energy consumption. Ceramic membranes provided poor COD removal (6% for 20 nm pores, with even lower removal for 0.1 μm and 5 μm) and were therefore excluded from further consideration. Foam fractionation demonstrated > 80% COD removal with minimal water loss and an estimated energy demand of only ~ 3 kWh per 450 L treated, making it the most promising option for meeting the design goals. It was selected for further experimental development.

**Table 2:** Performance of six physical-chemical batch experiments towards meeting Greywater design treatment goals using a simplified Greywater composition (300 Mg L^− 1^ SDS and 58.5 Mg L^− 1^ ammonium bicarbonate)

Technology Approach	Chemical / Energy Input	COD Removal (%)	kWh (Treated 450 L day^− 1^)	Water Recovery Potential	Potential to Meet Treatment Standards
Coagulation	0-500 mg L^− 1^ FeCl_3_	77	< 0.1	Medium	Low
Adsorption	0–5 g L^− 1^ PAC	99	< 0.1	High	Medium
Photocatalysis	5 g L^− 1^ TiO_2_; 7.2 J cm^− 2^	9	552	High	Low
Electrocatalysis	Dual BDD 36 mA cm^− 2^	67	57	High	Medium
Ceramic UF Separation	UF/20-nm at 2.1 bar	6	14	Medium	Low
Foam Fractionation	Air at 0.5 L min^− 1^	82	3	Medium/High	High

#### Factors influencing foam fractionation in column experiments

**Fig. 3:**
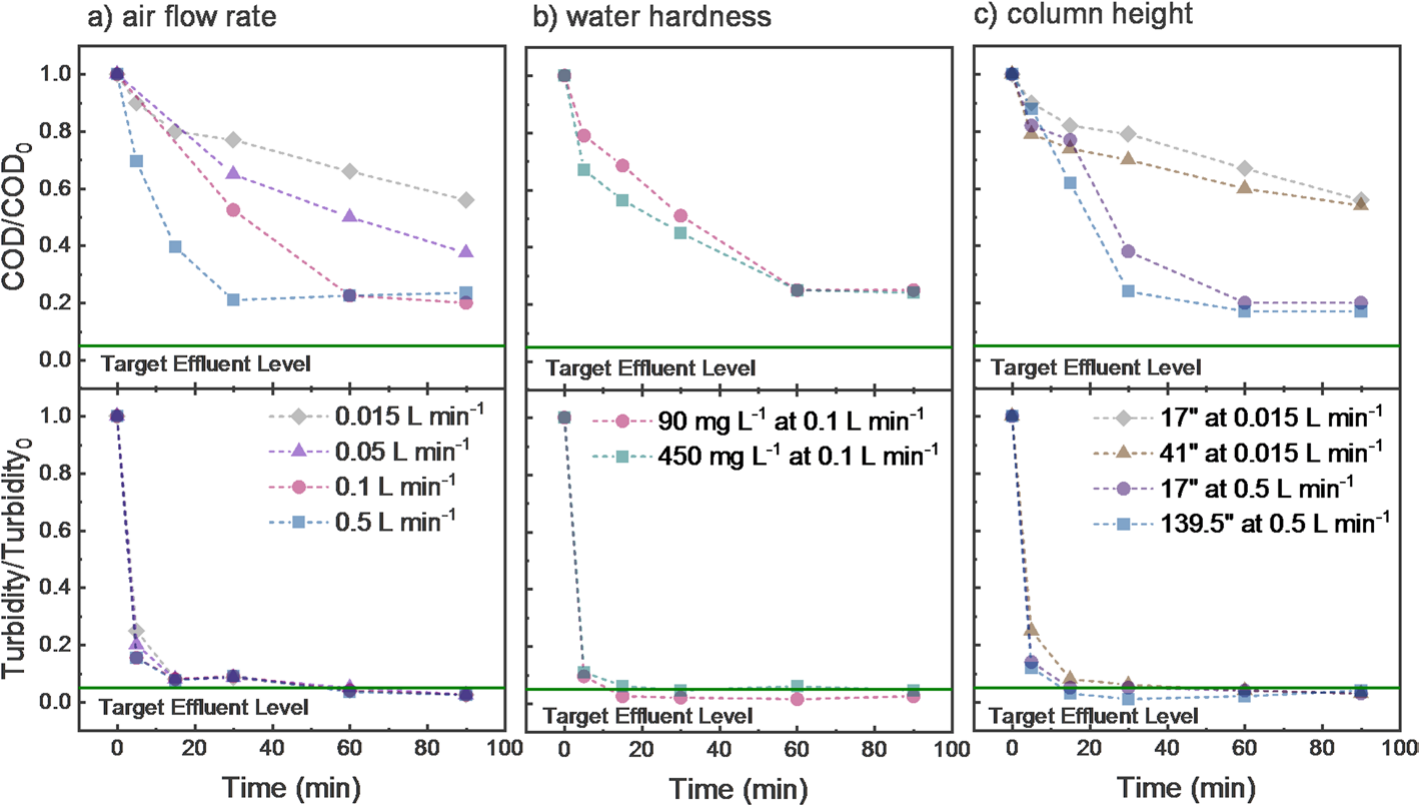
Fraction of COD and turbidity (NTU) remaining in liquid (non-foam) volumes of water during different durations of aeration to induce foaming for **a** different air flowrates (mL min^− 1^), **b** water hardness and **c** reactor column heights (17, 41, 139.5 in. and air flowrates) using a simplified greywater composition (300 mg L^− 1^ SDS and 58.5 mg L^− 1^ ammonium bicarbonate)

Foam fractionation experiments in both small and large columns (Figs. 1a and b) were conducted to assess the viability of this treatment approach. Since hard water can affect foaming behavior, the simplified water matrix (Table 2) was spiked with CaCO_3_ and subjected to foam fractionation. As shown in Fig. 3b, COD removal from the treated water gradually increased over 90 min of aeration, with little difference between 90 and 450 mg CaCO_3_ L^− 1^ hardness. Turbidity removal exceeded 94% within 30 min. Across the hardness range tested, foam fractionation performance remained stable. Additional tests (not shown) examined ionic strength effects. COD removal exceeded 80% when ionic strength was ≥ 8 mM NaCl (the baseline for synthetic greywater) but dropped to 40–80% at lower concentrations. This suggests performance may decline in very soft or low-conductivity water, rather than in hardwater conditions.

Column height was also evaluated, based on the idea that water might drain from foam in taller columns – allowing higher treated water recovery to be achieved. As shown in Fig. 3c, column height had no significant impact on COD or turbidity removal. However, the tallest system was deemed physically impractical, prompting the development of alternative configurations using shorter columns, foam separation, and external foam collapse chambers.

Surfactants facilitate bubble formation by orienting themselves at the air–water interface: the hydrophobic tail associates with the bubble surface, while the polar head remains in the aqueous phase. This behavior promotes efficient surfactant removal during foam generation. Similarly, particulates such as kaolin that contribute to turbidity become entrapped within the foam structure and are removed along with the separated foam. Overall, these batch experiments validated foam fractionation as a feasible treatment approach. Performance benchmarks were consistent with the literature, which typically reports ~ 85% COD removal from greywater using foam fractionation [28, 29].

#### Foam separation, collection and collapsing reactor (Gen1 Reactor)

Based upon the success in batch reactors, a Gen1 reactor was fabricated and operated which was capable of foam production and collection in one reactor with separate collapsing of the foam using pressurized air in a second reactor. This allowed for evaluation of a more integrated design, which could be operated in the future in a semi-batch mode. Additionally, this Gen1 reactor allowed evaluation of both COD or turbidity removal and water recovery. Therefore, semi-batch operation was investigated using 1.5 L volumes of greywater (Table 1) cycled into the aeration chamber (Fig. 1c) and samples collected from the bottom of the aeration chamber at various periods over 90 min.

**Fig. 4:**
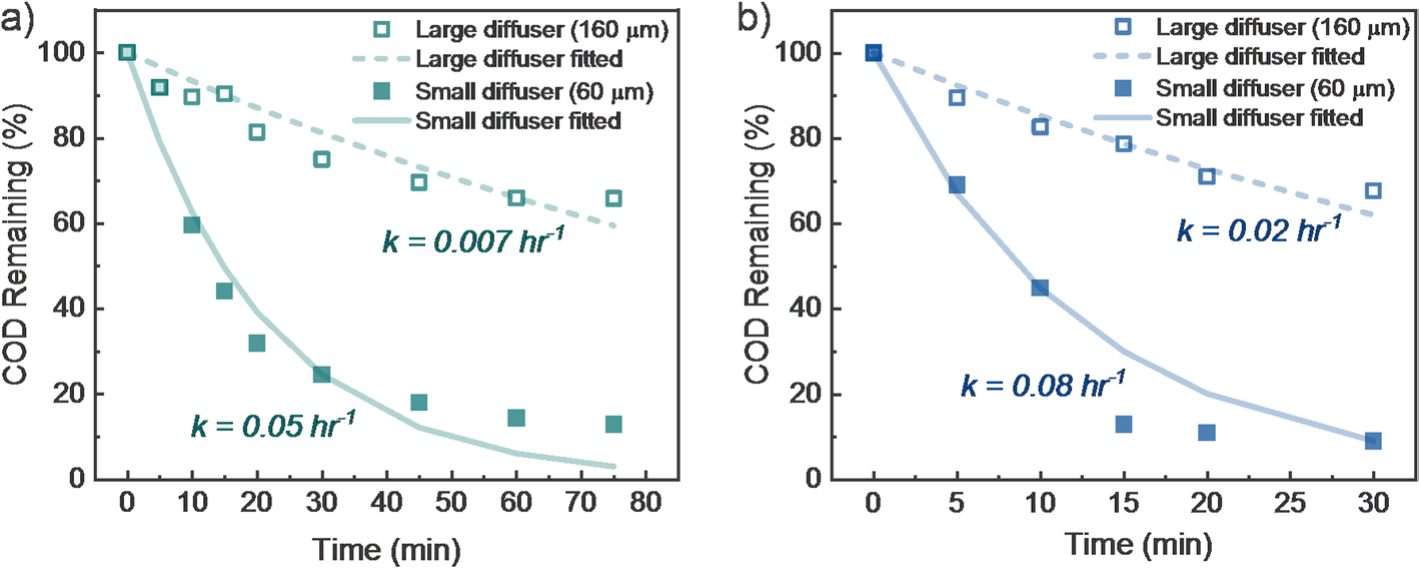
COD removal kinetics during foam fractionation experiments in the Gen1 reactor for two diffusers with 60–160 μm pores at **a** 0.5 L min^− 1^ and **b** 1.5 L min^− 1^ of continuous air flowrates. Symbols represent measured data and lines represent pseudo first order fits of the data with associated *k* values (hr^− 1^). Experiments were performed using the simplified greywater matrix

To evaluate the kinetics of COD removal during foam fractionation, samples were collected over time. Figure 4 shows the decline in COD concentration in the non-foam liquid phase over time for different diffuser sizes and air flowrates. The data fit well to a pseudo-first-order model:

2 $${\text{COD}}/{\text{CO}}{{\text{D}}_0}\,=\,{{\text{e}}^{ - \,{\text{kt}}}}$$

where *k* (hr⁻¹) is the rate constant. Smaller diffuser pores produced faster COD removal, likely due to the generation of smaller bubbles with greater surface area. Higher air flowrates also slightly improved COD removal, likely by increasing the number of bubbles formed per unit time. Overall, diffuser pore size had a stronger impact on COD removal kinetics than air flowrate.

**Fig. 5:**
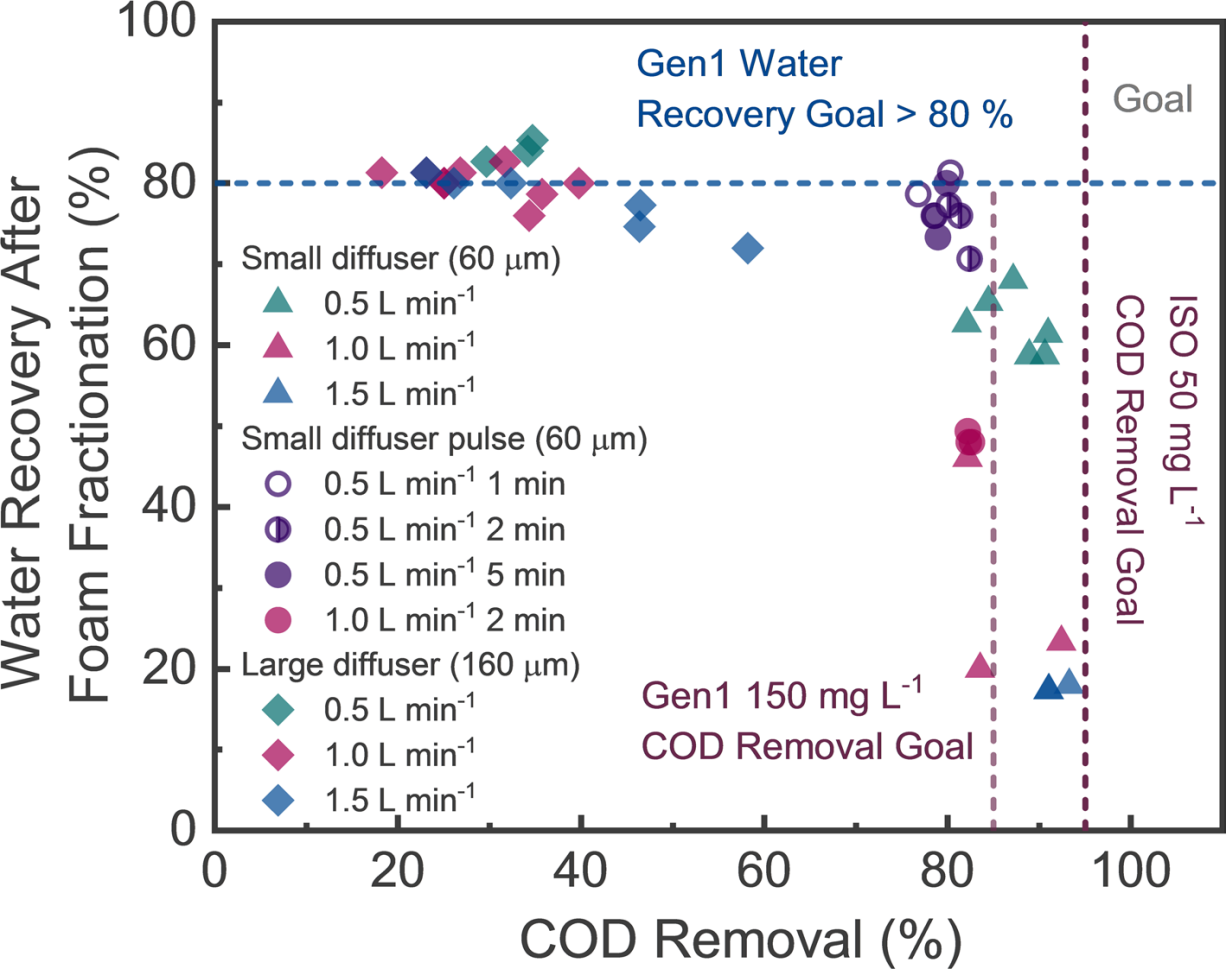
Experimental results of foam fractionation in the Gen1 reactor using the representative greywater matrix (Table 1) with smaller (60 μm) or larger (160 μm) pore size diffusers and different air flowrates (0.5 to 1.5 L min^− 1^) applied continuously or pulsed

Figure 4 demonstrates the potential for effective COD removal using the Gen1 reactor with a simplified greywater matrix. Subsequent experiments used a more complex matrix (Table 1) and tested three air flowrates (0.5 to 1.5 L min^− 1^). With the smaller 60 μm pore size diffuser, COD removal ranged from 82% to 92%, with minimal sensitivity to air flowrate. In contrast, the larger 160 μm diffuser achieved only 32–40% COD removal. Bubble size distributions varied by diffuser type and air flowrate. The small-pore diffuser generated bubbles of approximately 300–400 μm, 400–600 μm, and 600–1000 μm at flowrates of 0.5, 1.0, and 1.5 L min^− 1^, respectively. The large-pore diffuser produced larger bubbles: ~500–600 μm, 600–800 μm, and 700–1500 μm for the same respective flowrates. Experimental data are presented in Fig. 5. Water recovery results are discussed in Sect. 3.2.3.

To evaluate the adaptability of foam fractionation for handwashing station applications, triplicate experiments were conducted using a modified greywater formulation that included commercial liquid hand soap. The recipe used tap water spiked with kaolin, cellulose, and yeast extract in the same proportions as the original synthetic greywater but replaced the surfactant mixture with 3.6 g L^− 1^ of hand soap. Foam fractionation was performed at 1.0 L min^− 1^ using both 60 μm and 160 μm pore diffusers. System performance was comparable to that observed with the original synthetic greywater formulation. Statistical analysis showed no significant difference (*p*-value = 0.16 for the 60 μm diffuser; *p*-value = 0.30 for the 160 μm diffuser), supporting the null hypothesis that the groups were equivalent.

Surfactants are the primary contributors to COD in greywater. At the high COD levels typically present (~ 1000 mg L^− 1^), physical-chemical oxidation would require substantial energy (Table 2), far exceeding the performance target of 1 kWh per day for treating 450 L of greywater. Foam fractionation removes COD effectively because surfactants encapsulate air bubbles. The tendency of surfactants to self-organize and form micelles is often characterized by surface tension measurements, used to determine the critical micelle concentration (CMC). To better understand foam generation behavior, we examined the relationship between surfactant composition and foaming performance. This included evaluating individual surfactants, mixtures representative of complex greywater (Table 1), and changes in surfactant behavior over time during treatment with the Gen1 foam fractionation reactor.

**Fig. 6:**
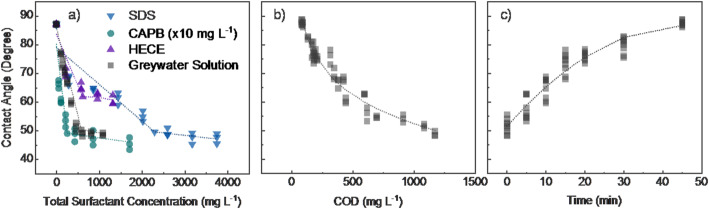
Surface contact angle measurements for **a** individual surfactants or synthetic greywater as a function of surfactant concentration and change the liquid phase surface contact angle measurement as a function of **b** treatment time and **c** residual COD concentration during treatment in the Gen1 reactor (60 μm diffuser at 1.0 L min^− 1^)

We hypothesized that once the critical micelle concentration (CMC) is reached, foaming diminishes because excess surfactants form micelles rather than stabilizing bubbles (see further details in Supplemental Information **Text S2**). For both individual and mixed surfactants, Fig. 6a shows that surface contact angle initially decreased (approaching that of ultrapure water, ~ 90–92°) at low concentrations, then plateaued beyond the CMC. Among the surfactants tested, SDS had the highest CMC, followed by HECE and CAPB. The CMC of the greywater mixture fell between those of the individual components. During foam fractionation in the Gen1 reactor, surface contact angle started low and gradually increased from ~ 50° to ~ 90–92° as treatment progressed (Fig. 6b), consistent with surfactant depletion. Correspondingly, Fig. 6c shows that COD concentration decreased over time, displaying a similar trend to the contact angle curve in Fig. 6a. Both COD removal and the rate of contact angle increase slowed with extended treatment time. These results support the hypothesis that surfactant behavior—particularly surface association at the air–water interface—dominates foaming and associated COD removal during foam fractionation. Therefore, the type of surfactants used in hand soaps and other detergents influences the effectiveness of foam fractionation. This suggests a promising strategy: designing cleaning agents with low critical micelle concentrations (CMC) to enhance COD removal in foam-based treatment systems.

#### Water recovery

In all Gen1 experiments, water recovery was quantified alongside COD removal. Figure 5 presents the results for different diffuser types and air flowrates. Water recovery generally exceeded 70% when COD removal was below 75%; however, achieving higher COD removal (> 75%) led to a decline in water recovery. Water recovery decreased when a larger volume of water, containing COD, overflowed into the foam collection chamber of the Gen1 system. To meet the treatment targets outlined in Fig. 5, we explored strategies to improve COD removal without sacrificing water recovery.

Visual observations during operation revealed that water was retained within the foam during continuous aeration and subsequently carried into the foam collection chamber—contributing to lower water recovery. To address this, we implemented pulse air addition, where aeration was alternated with resting periods, allowing water to drain from the foam before it overflowed (see Fig. 1c). To evaluate this, experiments were conducted using synthetic greywater at a 0.5 L min^− 1^ air flowrate. Aeration was applied for 2 min, followed by resting periods of 1, 2, or 5 min. As shown in Fig. 5, this approach increased water recovery by ~ 20% across all cases. However, COD removal decreased by ~ 10% for each of the pulsed conditions. The combined data in Fig. 5 fits to an empirical power function model:

3 $$Water{\text{ }}Recovery{\text{ }}\left( \% \right)\,=\,5.8{\text{ }}AF{R^{ - \,1.9}}+{\text{ }}0.46{\text{ }}DPS\,+\,20\left( {PLS} \right)$$

where AFR is the air flowrate (L min^− 1^), DPS is the average diffuser pore size (µm), and PLS is a binary variable (1 for pulsed aeration, 0 for continuous mode). The R^2^ for Eq. 3 is 0.90. While modest, these improvements helped bring the Gen1 system closer to our target performance (i.e., toward the upper-right corner of Fig. 5). Additionally, pulse aeration offers a secondary benefit—reducing compressor energy demand—which supports the system’s energy efficiency goals.

### Dual chamber reactor for foam production and collapse

Foam generated during fractionation in the Gen1 reactor was collected in a separate, isolatable chamber where it was collapsed for easier storage and potential surfactant recovery. Several foam collapse strategies were considered, but we selected **pressurization**, leveraging the existing air pump already used for bubble generation. Once foam entered the collection chamber, a valve isolated the chamber, and it was pressurized to ~ 1.4 bar—sufficient to collapse the foam into liquid waste. To enable continuous operation, the system used a single foam fractionation column paired with two parallel foam collection chambers, each capable of being isolated and pressurized. The system alternated between the two chambers, allowing for uninterrupted greywater treatment.

To demonstrate its operation, a representative treatment cycle at 0.5 L min^− 1^ with the 60 μm diffuser yielded the following results: 1.5 L of synthetic greywater (initial COD = 1000 mg L^− 1^) was processed. After treatment, 1 L of treated water remained in the aeration chamber with a COD of 170 mg L^− 1^. The remaining 0.5 L, collected in the foam collapse chambers, had a concentrated COD of 2660 mg L^− 1^. Treatment of this waste stream will be explored in future work. Multiple treatment cycles were run with consistent performance. While this system was not optimized to exactly meet the water recovery targets shown in Fig. 5, the Gen1 design successfully demonstrated proof-of-concept for continuous foam fractionation with integrated foam collapse.

### Electrochlorination before and after foam treatment

**Fig. 7:**
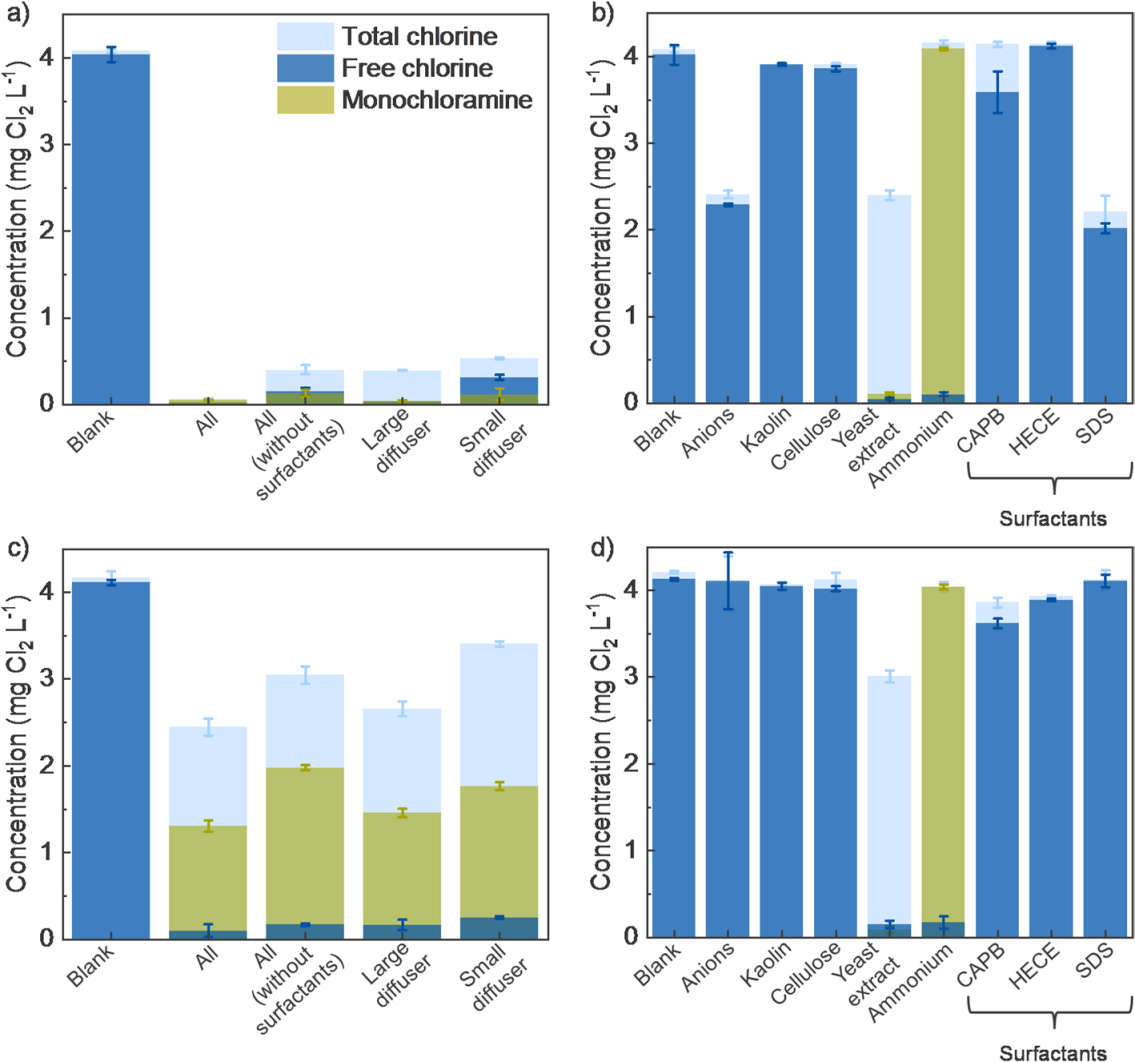
Electrochemical and Chemical Chlorination of Various Water Matrices. Total chlorine, free chlorine, and monochloramine concentrations after a single pass of the electrochemical cell of waters containing **a** only chloride (blank), all compounds present in greywater, all compounds except surfactants, greywater after treatment with large diffuser, and after treatment with small diffuser; **b** chloride and co-existing individual compounds with corresponding dosages found in greywater. The water samples were passed through the electrochemical flow cell at flow rate of 0.3 L min^− 1^ and under current of 0.2 A. Total chlorine, free chlorine, and monochloramine concentrations after manual addition of 4 mg L^− 1^ NaOCl in water containing **c** only chloride (blank), all compounds present in greywater, all compounds except surfactants, greywater after treatment with large diffuser, and after treatment with small diffuser; **d** chloride and co-existing individual compounds with corresponding dosages found in greywater

We found that surfactant removal is an essential step to ensure effective electrochemical generation of active chlorine species. As shown in Fig. 7a, after a single pass through the electrochemical cell with 0.8 s of hydraulic residence time, clear water containing only chloride (Cl^−^) as anions (i.e., blank) resulted in the formation of ~ 4 mg L^− 1^ of free chlorine species (Cl_2(aq)_/HOCl/OCl^−^). In contrast, water having all compounds present in untreated greywater (i.e., all) produced a negligible total chlorine concentration of near 0.06 mg L^− 1^ (Fig. 7a), which includes both free chlorine species and combined chlorine compounds (NH_2_Cl/NHCl_2_/NCl_3_). Electrochlorination of greywater matrix without surfactants (i.e., all (without surfactants)) led to a significant increase in total chlorine production, reaching up to 0.4 mg L^− 1^, consisting of 0.15 mg L^− 1^ of free chlorine and 0.13 mg L^− 1^ of monochloramine (NH_2_Cl). These results underscore the inhibitory nature and/or scavenging effect of organic surfactants on chlorine residuals. Foam separation process achieved high COD removal and effective water recovery. The fractioned water was then evaluated for its potential to generate aqueous chlorine using the electrochlorination process.

Single pass through the electrochemical cell of water after treatment with large diffuser (i.e., near 30% COD removal and 84% of water recovery) resulted in approximately 0.39 mg L^− 1^ of total chlorine generation (Fig. 7a), which is comparable to the chlorine yield from electrochlorination of greywater matrix without surfactants. However, negligible free chlorine and NH_2_Cl residuals were detected. In contrast, foam separation using a small diffuser (i.e., near 85% COD removal and 65% of water recovery) significantly increased total chlorine generation up to 0.53 mg L^− 1^, with 0.31 mg L^− 1^ of free chlorine and 0.12 mg L^− 1^ of NH_2_Cl (Fig. 7a). These results indicate that the small diffuser was more effective not only in removing surfactants but also in removing additional compounds present in greywater that inhibit chlorine generation and/or consume chlorine species. This is supported by the elevated total and free chlorine residuals observed after electrochlorination and chemical chlorination of greywater treated with the small diffuser compared to concentration of the chlorine residuals in greywater matrix without surfactants (Figs. 6a and c).

Previous work on disinfection using the same electrochemical system demonstrated more effective microorganisms’ inactivation using electrochlorination compared to chemical chlorination approach. Those results showed that *Ct* value (i.e., *C* (concentration of free chlorine) multiplied by *t* (contact time)) of 1.75 min-mg L^− 1^ was enough to ensure inactivation of 5.8 log of bacteriophage MS2 and 6.2 log of bacteria *E. coli*. The enhanced disinfection was attributed to high chlorine concentration and low pH near the anode surface [25]. Thus, free chlorine of 0.31 mg L^− 1^ generated after the treatment with small diffuser could potentially result in similar log removal of MS2 and *E. coli*, provided the contact time exceeds 6 min. Notably, in situ electrochlorination following foam removal with small diffuser can ensure secondary water disinfection, as it holds chlorine residual ≥ 0.2 mg L^− 1^.

To evaluate which species present in greywater are most influential on in situ electrochlorination, each compound was tested individually. Figure 7b demonstrates that among the tested species, anions (i.e., NO_3_^−^, H_2_PO_4_^−^, SO_4_^2−^, and HCO_3_^−^), yeast extract, ammonia (NH_4_^+^/NH_3_), CAPB (C_19_H_38_N_2_O_3_), and SDS (NaC_12_H_25_SO_4_) had a negative impact on chlorine residuals after electrochlorination.

The impact on active chlorine species availability can be associated with (i) chemical scavenging reactions that consume electrogenerated chlorine, and/or (ii) inhibition of chlorine electrogeneration due to competition for active sites on the anode surface. To elucidate the driving mechanism that diminishes the concentration of active chlorine species, chemical chlorination was conducted to ascertain the scavenging character of other matrix components. Figure 7c illustrates that higher concentrations of chlorine residuals were detected with the chemical addition of chlorine than during in situ electrochlorination. This indicates that species present in the greywater compete on the anode surface and inhibit chlorine production.

Comparison of Fig. 7b and d shows that anions present in water including SDS inhibit chlorine generation on the anode surface since no change in chlorine concentrations were observed during chemical chlorination with these anionic species present. The SDS dissociates in water and negatively charged C_12_H_25_SO_4_^−^ compete for active sites on the anode surface. The negative influence of coexisting anions on chlorine generation has been discussed elsewhere, where inorganic anions including SO_4_^2−^, ClO_3_^−^, NO_3_^−^, HCO_3_^−^, and H_2_PO_4_^−^ have inhibitory impact on chlorine generation due to adsorption on the anode surface and blocking active sites for chlorine generation [25, 26, 30–32].

In the case of yeast extract, NH_4_^+^/NH_3_, and C_19_H_38_N_2_O_3_, chlorine quenching occurs after chlorine being electrogenerated, as comparable results were obtained with homogeneous chemical chlorination (Fig. 7d). Ammonia does not inhibit electrochemical chlorine generation but increases chlorine demand [33]. The weight ratio of chlorine to ammonium as nitrogen in greywater (i.e., weight ratio of Cl_2_ to NH_4_^+^-N is 4 to 10.4) is well below the breakpoint chlorination (i.e., weight ratio of Cl_2_ to NH_4_^+^-N is 7.6 to 1.0), so expectedly no ammonium is completely oxidized to N_2_ gas, and only NH_2_Cl formed. While NH_2_Cl is weaker disinfectant than free chlorine species, NH_2_Cl is also valued as a disinfectant for its long lifetime in the water and reduced formation of regulated disinfection by-products such as trihalomethanes and haloacetic acids [34, 35].

**Fig. 8:**
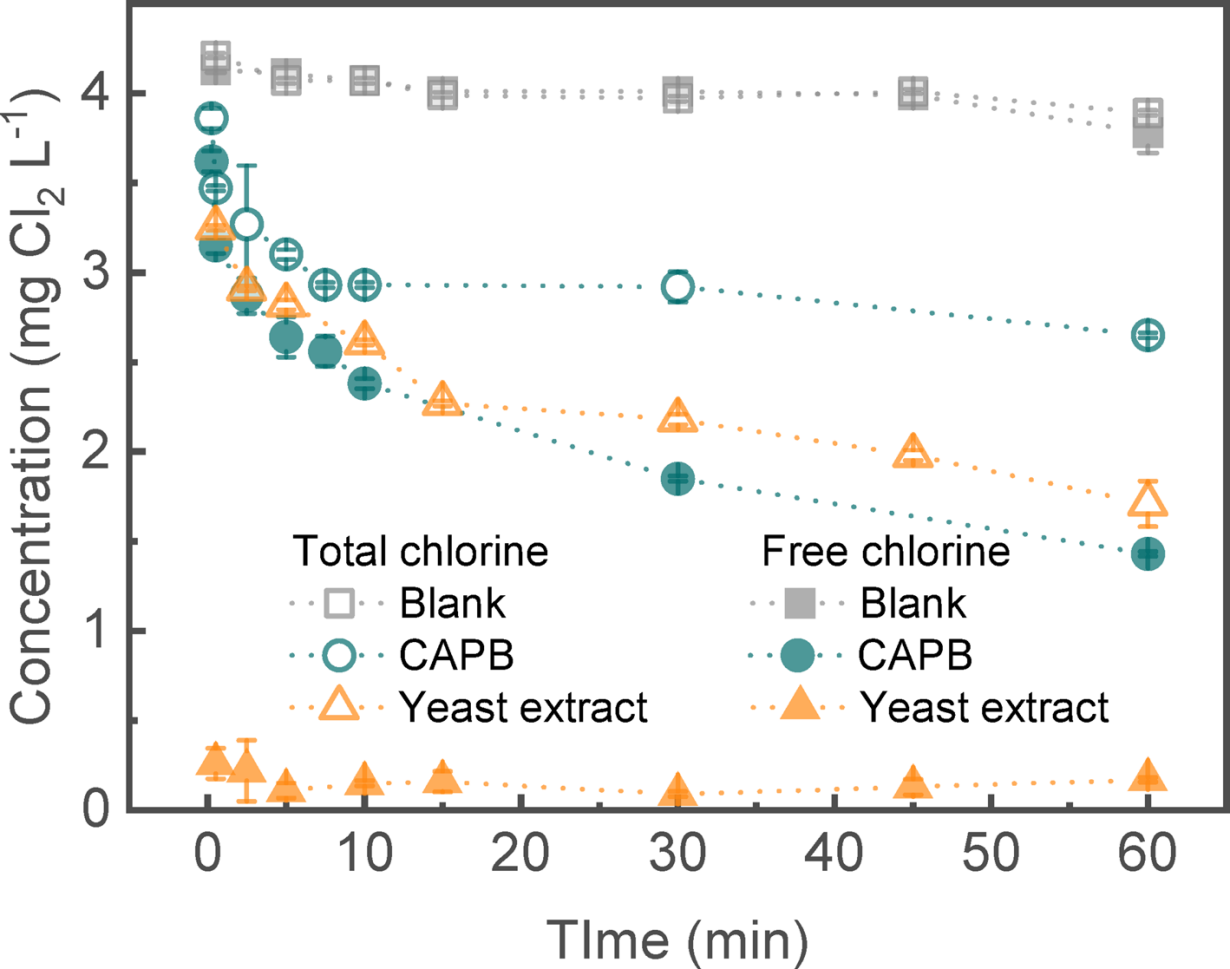
Residual chlorine over time. Total and free chlorine concentration after addition of 4 mg L^− 1^ NaOCl in waters containing chloride content (blank), CAPB, and yeast extract compounds

When analyzing chlorine demand with chlorine-scavenging compounds, it is important to remember that time plays a crucial role in residual chlorine since different organic compounds react with chlorine at different rates. Figure 8 illustrates that negligible differences were observed in total and free chlorine concentrations during chlorination of a blank solution. However, in the case of chlorination with zwitterionic C_19_H_38_N_2_O_3_ and yeast extract, the concentration of residual chlorine decreased over 60 min. In both cases, negligible amounts of NH_2_Cl were detected. The difference observed between total and free chlorine might be due to the reaction of nitrogen present in both compounds (e.g., *N* ≥ 10.5% in yeast extract) and chlorine. Notably, a substantial amount of chlorine was scavenged by CAPB in an hour. Removal of the surfactant with foam fractionation will prevent chlorine scavenging by C_19_H_38_N_2_O_3_.

Results on electrochlorination of various water matrices revealed that treatment of water following foam fractionation with the small diffuser is more effective than electrochlorination of greywater matrix without surfactants. This is an intriguing finding as it suggests that besides 85% of COD removal, the small diffuser also removes other species that result in chlorine demand and/or species that compete for active sites on the anode and inhibit electrochlorination. However, despite the observed higher chlorine residuals in greywater treated with the small diffuser, total chlorine level of 0.53 mg L^− 1^, including free chlorine of 0.31 and 0.12 mg L^− 1^ of NH_2_Cl, is not sufficient to ensure inactivation of chlorine-resistant microorganisms such as *Cryptosporidium* and *Giardia*. Integrating a pre-filtration step prior to electrochlorination might achieve several log reductions in *Cryptosporidium* and *Giardia* due to their large size, thereby leaving viruses as the primary microbial concern. Unlike *Cryptosporidium* and *Giardia*, viruses require significantly lower *Ct* values for inactivation with chlorine. Based on the Environmental Protection Agency (EPA) guidelines, *Ct* value of 3 min-mg L^− 1^ using free chlorine is sufficient to achieve 4 log inactivation against viruses at pH 6–9 and temperature of 20˚C [36]. Thus, extending the residence time to 10 min, free chlorine dose of 0.31 mg L^− 1^ might meet compliance with this regulatory target. Furthermore, even greater inactivation may be expected using electrochlorination, as demonstrated in our previous work that compared chemical and electrochemical disinfection [24]. Experiments with waters containing individual compounds present in greywater matrix further confirm inhibitory nature of anionic species on chlorine generation, as well as chlorine scavenging impact of nitrogen containing compounds. Therefore, it would be beneficial to consider a larger electrochemical cell design with an increased surface area of electrodes. This also would enable a larger electrode-electrolyte interface with high chlorine concentration and low pH, which might lead to enhanced disinfection. In addition, electrochemical cell operation strategy using pulsed current may help desorb inhibitory species from the anode surface, thereby freeing active sites for chloride oxidation. Additionally, achieving greater COD removal could further enhance chlorine generation. Thus, implementing another foam fractionation module might therefore significantly advance the subsequent electrochemical chlorination treatment.

## Summary and conclusions

Foam fractionation shows strong potential as a greywater treatment technology, capable of removing high levels of COD and turbidity with relatively low energy input. Water recovery can exceed 80% through the use of air pulsation, which allows foam films to drain before collection. However, its primary limitation is that COD removal depends on the foaming capacity of the greywater, which is governed by the solution’s critical micelle concentration (CMC).

In situ electrochlorination enabled by ambient chloride ion in greywater following foam fractionation—using a small-pore diffuser that achieved 87% COD removal (137 mg L^− 1^ remaining) and 68% water recovery—produced a residual chlorine level of 0.53 mg L^− 1^. This was higher than in trials without surfactants, suggesting that additional chlorine-scavenging compounds were removed during fractionation. Greywater composition significantly affects electrochlorination, as negatively charged species can block anode surfaces and ammonia or organics may scavenge free chlorine. Thus, optimizing operational parameters under various water conditions is essential. Additionally, since foam fractionation may contribute to microbial removal, the required chlorine dose for disinfection could potentially be reduced—though further studies are needed to quantify this effect.

Another important consideration is soap formulation and real-world greywater compositions. Many commercial soaps contain additives such as oils or antifoaming agents that may interfere with the foam fractionation process. For example, oils can cause foam destabilization by penetration into the foam film, which thins the bubble walls and humectants, which are often used in soaps and cosmetics to retain moisture in the skin, can sometimes negatively affect foam film properties, such as surface tension, in ways that promote bubble coalescence and destabilize the foam. To test real-world applicability, we developed a Gen2 semi-batch foam fractionation system (Figs. 1e and 2b) integrated with electrochlorination. This system was deployed at our university and tested with several low-cost commercial hand soaps and tap water. Each washing cycle used 200–300 mL of water (*via* footpad pump), producing greywater with initial COD levels of 1000–1500 mg L^− 1^. Treatment consistently achieved > 70% COD removal with high water recovery, and no adverse effects were observed in this real-use scenario. Future work will explore various failure modes, continued system automation, diffuser fouling, and microbial growth within the system in order to determine its stability. Additionally, the implementation of low-cost filters as secondary treatment and the further optimization of diffusers will be considered.

These results support the development of a low-energy, effective greywater treatment system based on foam fractionation. No chemical addition is required, and the process avoids sludge generation. The resulting liquid waste stream can be disinfected using the integrated electrochemical system, enabling safe disposal—or potentially allowing for surfactant or soap recovery. With further optimization, the system could reliably achieve < 50 mg L^− 1^ COD in treated water, maintain > 80% water recovery, and the integrated in situ electrochlorination using ambient chloride ion in greywater to ensure adequate disinfection prior to reuse.

## Supplementary Information

Below is the link to the electronic supplementary material.

Supplementary Material 1

## Data Availability

Data will be made available upon request to the corresponding author.
