# Supplementary material for: Towards low energy greywater treatment of surfactants and pathogens
Source: Discov Water. 2025 Oct 28;5(1):96. doi: 10.1007/s43832-025-00295-x (PMC12568821; doi:10.1007/s43832-025-00295-x)
Supplement: Supplementary file 1 — Supplementary Material 1 [file 43832_2025_295_MOESM1_ESM.docx]

**Supplementary Information**

**Towards Low-Energy Greywater Treatment of Surfactants and Pathogens**

Zachary Bogart, Aksana Atrashkevich, Jiripat Ananpattarachai, Shahnawaz Sinha, Sergi Garcia-Segura, Paul Westerhoff*

School of Sustainable Engineering and the Built Environment, Arizona State University, Tempe, Arizona, 85287-3005 USA

***Corresponding author:**

Paul Westerhoff Email: [p.westerhoff@asu.edu](mailto:p.westerhoff@asu.edu); Phone: 480-965-2885; orcid ID

0000-0002-9241-8759

**Additional Information on Bubble Formation and Separation during Foam Fractionation**

In order to understand influences on the foam fractionation process, it is important to discuss what factors influence bubble size and how they influence it since all of the parameters are essentially altering the bubble size of the foam. The pressure inside of a bubble is greater than the pressure around it. This pressure difference, called excess pressure, is described by Eqn-S1, where dp is excess pressure, $F_{g}$ is the force of gravity, and $a_{f}$ is the surface area [1].

$dp=\frac{F_{g}}{a_{f}}$ (Eqn-S1)

$F_{g}$ and $a_{f}$ can be calculated by Eqn-S2 and Eqn-S3 respectively, where R_d_ is the radius of the bubble, $\Delta\rho$ is the difference in density between the liquid in the film and the bulk solution, and $\gamma$ is the surface tension.

$F_{g}=\frac{4}{3}\pi R_{d}^{3}\gamma$ (Eqn-S2)

$a_{f}=\frac{2}{3}\pi R_{d}^{4}\frac{\Delta\rho}{\gamma}$ (Eqn-S3)

Combining and rearranging Eqns S1-S3 yields Eqn-S4, showing that the bubble size is related to the excess pressure and the surface tension.

$R_{d}=\frac{2\gamma}{\Delta\rho}$ (Eqn-S4)

This shows that the best way to control bubbles size is to control the excess pressure (the pressure through the diffuser) and the surface tension. More specifically, an increase in excess pressure or a decrease in surface tension will lead to a smaller bubble, while a decrease in excess pressure or an increase in surface tension leads to the formation of a larger bubble.

During foam fractionation a large amount of the initial chemical oxygen demand (COD) in the greywater is removed. This is because much of the COD in greywater comes from the surfactants, though there are other compounds, such as ammonium and cellulose, that contribute to the total COD. As the concentration of surfactants in the greywater decreases during foam fractionation, the contact angle, and by extension the surface tension, of the solution increases, as can be seen in Figure 3. This can theoretically continue until the surfactant concentration decreases below the CMC of the solution, at which point no significant foam generation can occur. The surface tension of the solution is of particular importance regarding water recovery. It is known that lower surface tensions result in smaller foam bubbles[2]. As foam is created, two distinct types of foam bubbles can be seen in the foam fractionation column. Near the bottom of the column, wet, spherical foam bubbles, called *kugelschaum*, form [3]. As the foam moves towards the top of the column, drainage occurs, causing the foam to have dry, polyhedral bubbles, called *polyederschaum[3]*. When smaller bubbles are created, there is a greater air-water interface surface area to volume ratio and a greater water content in the foam. This increase in the water to surfactant ratio in the wet foam leads to a higher ratio in the dry foam as well, which causes a decrease in water recovery and an increase in COD removal.

Ions in the water also have a significant effect on COD removal. The foam fractionator was run with varying levels of NaCl in solution such that the Ionic strength ranged from 4.8 mM to 11 mM. It was found that COD removal increased with increasing ionic strength until 7.9 mM, at which point the COD removal started to decrease. This is in agreement with Kumar, Rawat, and Ghosh who showed that, for various salts, surfactant removal from an aqueous solution via foam fractionation decreases with increasing salt concentration over the range of 10 mM to 100 mM [3]. There are two factors that have a role in the switch from the first stage when COD removal increases with ionic strength to the second stage where COD removal decreases with increasing ionic strength. The first factor is that as ionic strength increases the electric double layer (EDL) thickness decreases, increasing the likelihood of particles interacting with the surfactants and therefore increasing the COD removal. The second factor is that as EDL repulsion decreases, Van der Waals and capillary forces control the foam film thickness and make it thinner[4]. This decreases the water to surfactant ratio in the foam along with the COD removal.

In order to understand different methods for foam recovery and defoaming, it is necessary to know what affects a bubble’s film stability and understand what causes the film to rupture. The main aspects that govern the stability of surfactant bubbles are Gibbs free energy, van der Waals attractive forces, EDL repulsion forces, polymeric steric forces, and pressure, though other forces may have a role (J). For this section, the main items of focus are Gibbs free energy, van der Waals attractive forces, and pressure. There are two distinct steps that happen when a bubble ruptures naturally. The first step is film drainage. Film drainage happens due to forces such as gravity and van der Waals attraction[1]. There is a thickness that the film needs to reach before it can rupture, called the critical film thickness (h_c_), which is described by Eqn-S5, where AH is the Hamaker constant.

$h_{c}=0.267[{\frac{a_{f}{AH}^{2}}{6\gamma dp}]}^{1/7}$ Eqn-S5

The time it takes for the film to drain to the critical film thickness is dependent on various factors and, assuming the film is initially much thicker than the critical thickness and there is a constant force acting on the film, can be described by Eqn-S6 where t is time, u is viscosity, F is the constant force, and n is the number of immobile surfaces[1].

$t=\frac{3\mu a_{f}^{2}n^{2}}{16\pi Fh_{c}^{2}}$ Eqn-S6

The second phase that leads to film rupture is film deformation. Deformations can form on the film of the bubble, leading to areas with a higher Gibbs free energy. This increase is Gibbs free energy is offset by a decrease in energy from van der Waals forces, causing the film in the area to become thinner than the film around it. However, sometimes this decrease in van der Waals forces is greater than the increase in Gibbs free energy. This causes the deformation to increase in size and, if the film is at the critical thickness, potentially rupture the film. Between these two steps, the film draining to the critical film thickness is much slower and is therefore the rate limiting step [1].

**References**

1. Ghosh, P., *Colloid and Interface Science*. 2009: PHI Learning Pvt. Ltd. 519.

2. Kováts, P., D. Thévenin, and K. Zähringer, *Influence of viscosity and surface tension on bubble dynamics and mass transfer in a model bubble column.* International Journal of Multiphase Flow, 2020. **123**: p. 103174.

3. Radingoana, M.P., T. Dube, and D. Mazvimavi, *Progress in greywater reuse for home gardening: Opportunities, perceptions and challenges.* Physics and Chemistry of the Earth, Parts A/B/C, 2020. **116**: p. 102853.

4. Joye, J.L., G.J. Hirasaki, and C.A. Miller, *Dimple formation and behavior during axisymmetrical foam film drainage.* Langmuir, 1992. **8**(12): p. 3083-3092.
